# *Notes from the Field:* Three Human Rabies Deaths Attributed to Bat Exposures — United States, August 2021

**DOI:** 10.15585/mmwr.mm7101a5

**Published:** 2022-01-07

**Authors:** Amber Kunkel, Faisal S. Minhaj, Florence Whitehill, Connie Austin, Christine Hahn, Amanda J. Kieffer, Leila Mendez, Jael Miller, Leslie A. Tengelsen, Crystal M. Gigante, Lillian A. Orciari, Agam K. Rao, Ryan M. Wallace

**Affiliations:** ^1^Division of High-Consequence Pathogens and Pathology, National Center for Emerging and Zoonotic Infectious Diseases, CDC; ^2^Epidemic Intelligence Service, CDC; ^3^Illinois Department of Public Health; ^4^Idaho Department of Health and Welfare; ^5^Texas Department of State Health Services, Region 8, San Antonio, Texas; ^6^Lake County Health Department, Waukegan, Illinois; ^7^Texas Department of State Health Services, Region 6/5S, Houston, Texas.

During September 28–November 10, 2021, CDC confirmed three human rabies deaths in the United States, all in persons who did not seek postexposure prophylaxis (PEP) after bat exposures that occurred during August 2021. This increase in bat-associated human rabies deaths in the United States followed only three deaths during the previous 48 months. The cases during fall 2021 occurred in two adults and one child, all male, from Idaho, Illinois, and Texas. Initial symptoms included pain and paresthesia near the site of exposure progressing to dysphagia, altered mental status, paralysis, seizure-like activity, and autonomic instability. All three patients had recognized direct contact (e.g., bite or collision) with a bat approximately 3–7 weeks before symptom onset and died approximately 2–3 weeks after symptom onset. The deaths were associated with three bat species: *Lasionycteris noctivagans* (silver-haired bat), *Tadarida brasiliensis* (Mexican free-tailed bat), and *Eptesicus fuscus* (big brown bat) (Figure). All three species are common in the United States and have been implicated in previous rabies cases. One patient submitted the bat responsible for exposure for testing but refused PEP, despite the bat testing positive for rabies virus, due to a long-standing fear of vaccines. The other two patients did not realize the risk for rabies from their exposures, either because they did not notice a bite or scratch or did not recognize bats as a potential source of rabies. Case and contact investigations were led by the appropriate state and local health departments, and all human laboratory testing occurred at CDC. This activity was reviewed by CDC and conducted consistent with applicable federal law and CDC policy.*

**FIGURE:**
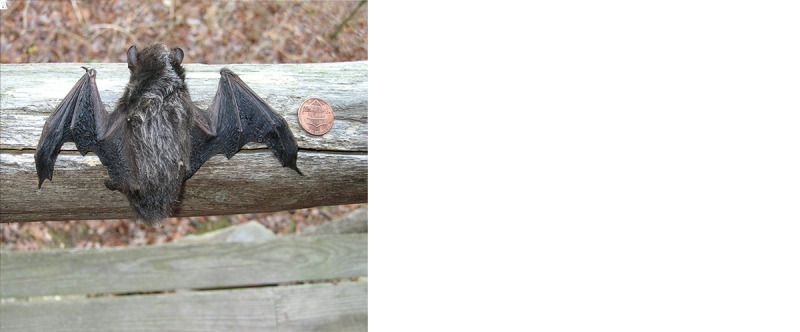
Three bat species A) *Eptesicus fuscus* (big brown bat), B) *Lasionycteris noctivagans* (silver-haired bat), and C) *Tadarida brasiliensis* (Mexican free-tailed bat) implicated in three human exposures — United States, August 2021 Photo A/unidentified patient; Photo B/Mark Mayfield; Photo C/Stephen Gergeni.

Rabies is a zoonotic disease transmitted primarily through virus-laden saliva from the bite of an infected mammal. The typical incubation period from exposure to symptom onset is 3–12 weeks. Rabies is nearly always fatal once symptoms develop but nearly always preventable when PEP is administered in accordance with the recommendations of the Advisory Committee on Immunization Practices.^†^ During 1960–2018, approximately 70% of 89 human rabies cases acquired in the United States were caused by exposures to bats (*1*). Although human rabies deaths in the United States are rare, rabid animals and rabies exposures are relatively common (*2*). Since 2014, all states except Hawaii have reported rabid bats. In 2020, public health programs tested approximately 24,000 bats for rabies, 1,401 (5.8%) of which were confirmed positive. CDC estimates that 60,000 persons each year receive rabies PEP following animal exposures (*3*), approximately two-thirds of these may be attributed to bats, depending on the local rabies epidemiology (*4*).

Preventing transmission of rabies from bats to humans can be accomplished by 1) avoiding contact with bats, 2) safely capturing and testing bats implicated in human exposures, and 3) seeking rapid evaluation for PEP when direct bat contact occurs and rabies cannot be ruled out. Two of the bat-associated cases in fall 2021 were considered avoidable exposures: one was attributed to a bat roost in the patient's home, the other to the patient picking up the bat with his bare hands. Safely excluding bats from homes and instructing persons not to touch bats can prevent rabies exposures.^§^ Two patients released the bat after contact had occurred rather than capturing it for testing. When a person has known or potential (e.g., while sleeping) contact with a bat, it should be safely captured,^¶^ if possible, and tested at a qualified laboratory. Timely bat rabies testing can save lives by ensuring persons at highest risk for rabies receive PEP, as well as reduce the cost, time, and resources associated with unnecessary PEP. PEP should be considered for any person who has direct contact with a bat unless the bat tests negative for rabies or public health officials can be reasonably certain there is no exposure risk.

Bats are ecologically critical species with seasonal activity patterns. Although bat activity is reduced in winter months, increased human-bat contacts often occur again in late spring to early fall (*5*). Avoiding contact with bats is the best way to protect both bat and human health. When human-bat contact is unavoidable, bat rabies testing and PEP are highly effective strategies to save human lives.

## Footnotes

* 45 C.F.R. part 46.102(l)(2), 21 C.F.R. part 56; 42 U.S.C. Sect. 241(d); 5 U.S.C. Sect. 552a; 44 U.S.C. Sect. 3501 et seq.

†
https://www.cdc.gov/mmwr/preview/mmwrhtml/rr5902a1.htm

§
https://www.cdc.gov/rabies/bats/contact/home.html

¶ https://www.cdc.gov/rabies/bats/contact/capture.html
